# Supplementary material for: Modelling Skylarks (Alauda arvensis) to Predict Impacts of Changes in Land Management and Policy: Development and Testing of an Agent-Based Model
Source: PLoS One. 2013 Jun 6;8(6):e65803. doi: 10.1371/journal.pone.0065803 (PMC3675089; doi:10.1371/journal.pone.0065803)
Supplement: Supporting Information S4 — The skylark ODdox as a zipped archive. (ZIP) [file pone.0065803.s004.zip › Skylark_ODdox/class_raster_map.html]

ALMaSS Skylark ODdox: RasterMap Class Reference


ALMaSS Skylark ODdox
 2.0


- Main Page
- Related Pages
- Classes
- Files

- Class List
- Class Index
- Class Hierarchy
- Class Members

Public Member Functions |
Private Attributes

RasterMap Class Reference

`#include <rastermap.h>`

List of all members.

|  |  |
| --- | --- |
| Public Member Functions | |
| int | Get (int a\_x, int a\_y) |
| char \* | GetID (void) |
| int \* | GetMagicP (int a\_x, int a\_y) |
| void | Manipulation1 () |
| int | MapHeight (void) |
| int | MapWidth (void) |
| void | Put (int a\_x, int a\_y, int a\_elem) |
|  | RasterMap (const char \*a\_mapfile) |
|  | ~RasterMap (void) |

|  |  |
| --- | --- |
| Private Attributes | |
| int | m\_height |
| char | m\_id [12] |
| int \* | m\_map |
| int | m\_width |
| int | m\_x |
| int | m\_y |

---

## Constructor & Destructor Documentation

|  |  |  |  |  |  |
| --- | --- | --- | --- | --- | --- |
| RasterMap::RasterMap | ( | const char \* | *a\_mapfile* | ) |  |

References cfg\_manipulation1\_on, g\_msg, m\_polymapping, CfgBool::value(), MapErrorMsg::Warn(), WARN\_FATAL, and WARN\_FILE.

{

FILE \* IFile;

char error\_num[20];

int lastref = -9999;

IFile = fopen(a\_mapfile, "rb" );

if ( !IFile ) {

g\_msg->Warn(WARN\_FILE, "RasterMap::RasterMap(): Unable to open file",

a\_mapfile );

exit(1);

}

fread( m\_id, 1, 12, IFile );

fread( &m\_height, 1, sizeof(int), IFile );

m\_width = m\_height;

m\_map = (int\*)malloc(sizeof(int)\* m\_width\*m\_height);

if ( m\_map == NULL ) {

g\_msg->Warn(WARN\_FATAL,

"RasterMap::RasterMap(): Out of memory.", "" );

exit(1);

}

fread( m\_map, 1, sizeof(int)\*m\_width\*m\_height, IFile );

m\_x = m\_width;

m\_y = m\_height;

for (int x=0; x<m\_x; x++ ) {

for (int y=0; y<m\_y; y++ ) {

int i = Get( x, y );

if ( i<0 || i> 250000 ) {

// This fills in holes caused by -9999, but if two consequetive holes are found, then it will exit

i = lastref;

if ( i<0 || i> 250000 ) {

sprintf( error\_num, "%d", Get( x, y ) );

g\_msg->Warn(WARN\_FILE, "RasterMap::RasterMap(): Unknown polygon map number (could be a hole):", error\_num );

exit(1);

}

Put( x, y , i);

}

lastref = i;

if ( m\_polymapping[ Get( x, y ) ] == -1 ) {

// Tripping out here means that the lsb file has a polygon that is not

// listed in the polygonrefs file

sprintf( error\_num, "%d", Get( x, y ) );

g\_msg->Warn(WARN\_FILE, "RasterMap::RasterMap():"

" Unknown polygon ref number:", error\_num );

exit(1);

}

}

}

fclose( IFile );

if (cfg\_manipulation1\_on.value()) {

Manipulation1();

exit(10);

}

}

|  |  |  |  |  |  |
| --- | --- | --- | --- | --- | --- |
| RasterMap::~RasterMap | ( | void |  | ) |  |

{

free( m\_map );

}

---

## Member Function Documentation

|  |  |  |  |
| --- | --- | --- | --- |
| int RasterMap::Get | ( | int | *a\_x*, |
|  |  | int | *a\_y* |
|  | ) |  |  |

inline

References g\_msg, MapErrorMsg::Warn(), and WARN\_BUG.

Referenced by Landscape::BorderStep(), Landscape::BorderTest(), Landscape::ChangeMapMapping(), Landscape::CountMapSquares(), Landscape::FindValidXY(), Landscape::OrchardBorderAdd(), Landscape::PolysValidate(), Landscape::SetPolyMaxMinExtents(), Landscape::StepOneValid(), Landscape::SupplyCountryDesig(), Landscape::SupplyDeadBiomass(), Landscape::SupplyElementSubType(), Landscape::SupplyElementType(), Landscape::SupplyElementTypeCC(), Landscape::SupplyFarmIntensity(), Landscape::SupplyFarmOwner(), Landscape::SupplyFarmOwnerIndex(), Landscape::SupplyFarmType(), Landscape::SupplyGrazingPressure(), Landscape::SupplyGreenBiomass(), Landscape::SupplyHasTramlines(), Landscape::SupplyInsects(), Landscape::SupplyJustSprayed(), Landscape::SupplyLAGreen(), Landscape::SupplyLastTreatment(), Landscape::SupplyLATotal(), Landscape::SupplyPolyRef(), Landscape::SupplyPolyRefCC(), Landscape::SupplyPolyRefIndex(), Landscape::SupplyTrafficLoad(), Landscape::SupplyVegAge(), Landscape::SupplyVegBiomass(), Landscape::SupplyVegCover(), Landscape::SupplyVegDensity(), Landscape::SupplyVegDigestability(), Landscape::SupplyVegHeight(), Landscape::SupplyVegPatchy(), Landscape::SupplyVegType(), Landscape::SupplyWeedBiomass(), Pesticide::TwinMapSpray(), and Landscape::UMarginTest().

{

if (a\_x<0 || a\_x>=m\_width ||

a\_y<0 || a\_y>=m\_height ) {

g\_msg->Warn( WARN\_BUG,

"RasterMap::Get(): Coordinates out of range!", "" );

exit(1);

}

return m\_map[ a\_y \* m\_width + a\_x ];

}

|  |  |  |  |  |  |
| --- | --- | --- | --- | --- | --- |
| char\* RasterMap::GetID | ( | void |  | ) |  |

inline

Referenced by Landscape::DumpMap().

{ return m\_id; }

|  |  |  |  |
| --- | --- | --- | --- |
| int \* RasterMap::GetMagicP | ( | int | *a\_x*, |
|  |  | int | *a\_y* |
|  | ) |  |  |

inline

Referenced by Landscape::DumpMap(), Landscape::hb\_Add(), and Landscape::SupplyMagicMapP().

{

return &m\_map[ a\_y \* m\_width + a\_x ];

}

|  |  |  |  |  |
| --- | --- | --- | --- | --- |
| void RasterMap::Manipulation1 | ( |  | ) |  |

References cfg\_rastermapmanipmultiply, and CfgInt::value().

{

FILE\* o1;

FILE\* o2;

o1=fopen("RasterDump1.lsb","wb");

o2=fopen("RasterDump2.lsb","wb");

char \* l\_id = m\_id;

int new\_height = m\_height\*2;

fwrite( l\_id, 1, 12, o1 );

fwrite( & new\_height, 1, sizeof( int ), o1 );

for (int y=0; y< m\_width; y++ ) {

for ( int x = 0; x < m\_width ; x++ ) {

int l\_poly = Get(x,y);

fwrite( & l\_poly, 1, sizeof( int ), o1 );

fwrite( & l\_poly, 1, sizeof( int ), o1 );

}

for ( int x = 0; x < m\_width ; x++ ) {

int l\_poly = Get(x,y);

fwrite( & l\_poly, 1, sizeof( int ), o1 );

fwrite( & l\_poly, 1, sizeof( int ), o1 );

}

}

int multiply=cfg\_rastermapmanipmultiply.value();

new\_height = m\_height\*multiply;

fwrite( l\_id, 1, 12, o2 );

fwrite( &new\_height, 1, sizeof( int ), o2 );

for (int m=0; m<multiply; m++) {

for (int y=0; y< m\_width; y++ ) {

for (int mm=0; mm<multiply; mm++) {

for ( int x = 0; x < m\_width ; x++ ) {

int l\_poly = Get(x,y);

fwrite( & l\_poly, 1, sizeof( int ), o2 );

}

}

}

}

fclose( o1 );

fclose( o2 );

}

|  |  |  |  |  |  |
| --- | --- | --- | --- | --- | --- |
| int RasterMap::MapHeight | ( | void |  | ) |  |

inline

Referenced by Landscape::BorderStep(), Landscape::BorderTest(), Landscape::ChangeMapMapping(), Landscape::CountMapSquares(), Landscape::FindValidXY(), Landscape::hb\_Add(), Pesticide::Pesticide(), Landscape::PolysValidate(), Landscape::SetPolyMaxMinExtents(), Landscape::StepOneValid(), Landscape::SupplySimAreaHeight(), and Landscape::UMarginTest().

{ return m\_height; }

|  |  |  |  |  |  |
| --- | --- | --- | --- | --- | --- |
| int RasterMap::MapWidth | ( | void |  | ) |  |

inline

Referenced by Landscape::BorderStep(), Landscape::BorderTest(), Landscape::ChangeMapMapping(), Landscape::CountMapSquares(), Landscape::FindValidXY(), Landscape::hb\_Add(), Pesticide::Pesticide(), Landscape::PolysValidate(), Landscape::SetPolyMaxMinExtents(), Landscape::StepOneValid(), Landscape::SupplySimAreaWidth(), and Landscape::UMarginTest().

{ return m\_width; }

|  |  |  |  |
| --- | --- | --- | --- |
| void RasterMap::Put | ( | int | *a\_x*, |
|  |  | int | *a\_y*, |
|  |  | int | *a\_elem* |
|  | ) |  |  |

inline

Referenced by Landscape::BeetleBankAdd(), Landscape::BorderScan(), Landscape::BorderScan2(), Landscape::ChangeMapMapping(), and Landscape::UnsprayedMarginScan().

{ m\_map[ a\_y \* m\_width + a\_x ] = a\_elem; }

---

## Member Data Documentation

int RasterMap::m\_height

private

char RasterMap::m\_id[12]

private

int\* RasterMap::m\_map

private

int RasterMap::m\_width

private

int RasterMap::m\_x

private

int RasterMap::m\_y

private

---

The documentation for this class was generated from the following files:

- rastermap.h
- rastermap.cpp


- RasterMap
- Generated on Thu Jan 10 2013 13:15:37 for ALMaSS Skylark ODdox by
   1.8.1.1
